# Polymeric curcumin nanoparticles by a facile in situ method for macrophage targeted delivery

**DOI:** 10.1002/btm2.10112

**Published:** 2018-11-05

**Authors:** Priyanka S. Jahagirdar, Pramod K. Gupta, Savita P. Kulkarni, Padma V. Devarajan

**Affiliations:** ^1^ Dept. of Pharmaceutical Sciences and Technology Institute of Chemical Technology Matunga Mumbai, MH India; ^2^ Radiation Medicine Centre Bhabha Atomic Research Centre Parel Mumbai, MH India

**Keywords:** Curcumin, *in situ*, nanoparticles, macrophages, nanoprecipitation, intracellular uptake, Box Behnken

## Abstract

Targeting macrophages is a promising strategy for improved therapy of intracellular infections as macrophages exhibit rapid phagocytosis of particles >200 nm. Entrapment of Curcumin (CUR) in nanocarriers could provide bioenhancement and macrophage targeting. We present a simple and facile *in situ* nanoprecipitation approach for instantaneous and on‐site generation of curcumin nanoparticles (ISCurNP). ISCurNP optimised by Box‐Behnken design exhibited average size of 208.25 ± 7.55 nm and entrapment efficiency of 90.16 ± 1.17%. Differential scanning calorimetry and X‐Ray diffraction confirmed amorphization of CUR in ISCurNP. Sustained release was observed over 72 hr *in vitro* at lysosomal pH 4.5. Rapid and high uptake in RAW 264.7 macrophages was confirmed by flow cytometry and high performance liquid chromatography. Confocal microscopy established localisation of ISCurNP in lysosomal compartment. The facile *in situ* nanoprecipitation method provides simple, scalable technology to enable macrophage targeted delivery of CUR, with great promise for improved therapy of intracellular infections.

## 1 INTRODUCTION

Curcumin (CUR), a natural polyphenol has generated tremendous excitement as a nutraceutical due to its multifarious pharmacological activities.^1^, ^2^ The anticancer^3^, ^4^, ^5^, ^6^ and anti‐inflammatory^7^, ^8^ properties of CUR have been widely explored. CUR, an established anti‐infective for eons, is currently being extensively researched for antibacterial including antimycobacterial, antiviral, and antifungal activity.^9^, ^10^, ^11^ Despite the excellent therapeutic potential, a considerable impediment in exploitation of CUR as a therapeutic agent is its hydrophobic nature and consequently poor aqueous solubility.^12^

Nanonization of particles has proven to be a competent approach to enhance solubility and bioavailability of hydrophobic poorly soluble drugs.^13^, ^14^ Furthermore, such drugs when entrapped in nanocarriers with size titrated in the range of 200–600 nm can aid targeting to the reticuloendothelial system (RES) organs and cells, especially macrophages, the primary site of various intracellular infections.^15^, ^16^, ^17^, ^18^ Macrophages constitute defense system of the body and exhibit rapid phagocytosis of particles >200 nm. Targeting the macrophages by nanocarriers thus provides a viable approach to restrict growth and survival of the organisms.

Conventional nanotechnology approaches are based on multiple steps and dictate the need for sophisticated equipment. Furthermore, isolation of nanoparticles (NPs) prepared and their stabilization by freeze drying also pose significant scale‐up challenges, constituting major hurdles in the development and translation of nano drug delivery systems.^19^ In contrast, in situ nanotechnology presents a method wherein generation of NPs is achieved by simple addition of a monophasic preconcentrate comprising drug, carrier (polymer/lipid), and stabilizers to an aqueous phase. Our group has reported in situ nanotechnology as a facile method for instantaneous and on‐site generation of NPs to enable point of care application.^20^, ^21^, ^22^ A distinct advantage of in situ technology is total bypass of the technological hurdles and stability issues associated with conventional NP fabrication methods. First generation in situ nanotechnology relies on dilution of monophasic preconcentrates with an aqueous media like potable drinking water just prior to oral administration or dextrose/dextrose saline injection just prior to intravenous injection. Enhanced oral bioavailability was demonstrated from a self nanoprecipitating preconcentrate of tamoxifen citrate, which on dilution with aqueous media resulted in the formation of polymeric tamoxifen NPs with ~85% entrapment efficiency and average particle size <250 nm.^20^ An in situ hybrid nanoparticulate drug delivery system of nevirapine designed for simultaneous targeting of multiple reservoirs in HIV exhibited >90% drug entrapment in a mixture of solid lipid NPs and micelles which was instantaneously generated when the preconcentrate was diluted with isotonic dextrose injection. Following intravenous administration in Sprague Dawley rats, the heterogenous nanosystem revealed high drug accumulation in the RES organs of liver and spleen and also the brain, which represents a remote HIV reservoir.^21^

Second generation in situ nanotechnology can additionally enable passive targeting through stealth or active targeting enabled by inclusion of a receptor ligand. Herein, the aqueous phase is provided along with the preconcentrate and comprises the stealth agent or receptor ligand. An in situ primaquine nanocarboplex anchored with the asialoglycoprotein receptor ligand pullulan was successfully designed for targeting to hepatocytes. The nanocarboplex revealed an average size of ~250 nm with >70% complexation. Active targeting to hepatocytes was confirmed by enhanced hepatocyte accumulation coupled with a very high hepatocytes: parenchymal cells ratio of ~75:25.^22^

In the present study, first generation in situ nanotechnology approach has been exploited as a practical approach for the design of in situ polymeric curcumin NPs (ISCurNP) with the following objectives: (a) Optimization of stable and reproducible ISCurNP by the facile in situ method (b) Evaluation of macrophage uptake and subcellular localization of ISCurNP in the RAW 264.7 murine macrophage cell line, as a first step in exploiting ISCurNP as a targeted nano delivery system for intracellular infections.

## 2 EXPERIMENTAL SECTION

### 2.1 Materials

Curcumin of 99% purity was a kind gift from Laurus Labs Limited, India. Resomer 502 Poly (lactide‐co‐glycolide) comprising of lactide:glycolide 50:50 ester terminated, *M*_w_ 7,000–17,000 was generously gifted by Evonik India Pvt. Ltd. Soluplus® and Kolliphor P188 were a gift from BASF India. *N*,*N*‐dimethylacetamide was purchased from SD Fine Chemicals whereas HPLC solvents methanol and acetonitrile were procured from Thermo Scientific. Dulbecco's modified eagle medium and Fetal bovine serum were purchased from Gibco while Prolong® Gold Antifade Reagent with DAPI and Lysotracker^TM^ Red were bought from Invitrogen. MTT (3‐[4,5‐Dimethylthiazol‐2‐yl]‐2,5‐Diphenyltetrazolium Bromide) and cell culture grade Dimethyl sulfoxide were purchased from Sigma Aldrich. All other reagents and chemicals used were of analytical grade.

### 2.2 Solubility

Solubility of CUR was determined in various nonaqueous solvents like propylene glycol, *N*,*N*‐dimethylacetamide (DMA), Transcutol HP, polyethylene glycol 300, and polyethylene glycol 400. Briefly, excess CUR was added to 1 ml of solvent by vortex mixing and intermittent bath sonication for 20 min and equilibrated at room temperature for 48 hr. Equilibrated samples were centrifuged at 16,350 rcf for 20 min and the supernatant was analyzed for CUR by UV spectrophotometry at λmax 425 nm.

Approximate solubility of Kolliphor P188, Soluplus® and Poly lactide‐co‐glycolide (PLGA) in DMA was determined by the incremental addition method. Briefly, 5 mg increments of PLGA/Soluplus® were added to 1 ml of DMA with intermittent vortexing and sonication, till no more sample dissolved and a slight excess remained. All the experiments were performed in triplicate.

### 2.3 Fabrication of ISCurNP

#### 2.3.1 Preconcentrate

Preconcentrate comprised of CUR (10 mg), Kolliphor P188/Soluplus® (10–20 mg), and PLGA (10–30 mg) dissolved in DMA (0.5–1 ml) by bath sonication for 5 min to obtain a clear solution.

#### 2.3.2 ISCurNP

Preconcentrates were added to filtered distilled water for instantaneous formation ISCurNP.

### 2.4 Optimization of ISCurNP

#### 2.4.1 Preliminary screening

Preliminary screening was carried out using the one variable at a time (OVAT) approach. Preconcentrates with varying amount of Kolliphor P188/Soluplus®, PLGA, and DMA were prepared. ISCurNP was generated by adding the preconcentrate to filtered distilled water and the critical variables and working concentration range influencing formation of ISCurNP were arrived at.

#### 2.4.2 Design of experiment: Box–Behnken design

ISCurNP was optimized by applying Box–Behnken design. Independent variables selected were concentration of Soluplus® (X1), concentration of PLGA (X2), and volume of DMA (X3) in the preconcentrate and the dependent variables (responses) were particle size (PS; Y1), and percent entrapment efficiency (%EE; Y2) of the generated ISCurNP. Curcumin concentration (10 mg) and aqueous dilution volume (10 ml) were maintained constant. The independent variables selected based on preliminary screening were assigned values of −1, 0 and +1 for low, middle, and high levels respectively (Supporting Information Table S1). Experimental design and analysis was performed using Minitab 17 (licensed version). A total of 15 runs were generated by the software. All batches were prepared in triplicate and the responses were recorded to generate the polynomial equations and response surface graphs. Three test batches were generated using the desirability function and the dependent variables were predicted. The actual data for the predicted batches was obtained through experimentation. Closeness of the predicted data was compared with the experimental values obtained by applying *t*‐test.

### 2.5 Evaluation of preconcentrate

#### 2.5.1 Clarity

Preconcentrate was visually evaluated against light and dark background for presence of foreign matter or undissolved components.

#### 2.5.2 Drug content

CUR content was evaluated by reverse phase high performance liquid chromatography (RP HPLC). RP HPLC analysis was performed at 25 °C using Kromasil column (250 × 4.6 mm, 5 μ) on Jasco LC 2000 (Jasco, Japan) fitted with UV detector (λ_max_ of 425 nm). Isocratic elution conditions were maintained with mobile phase comprising of potassium phosphate buffer (50 mM): Methanol:Acetonitrile (40:30:30) pH adjusted to 3 with orthophosphoric acid at a flow rate of 1.4 ml/min. The preconcentrate (0.75 ml) was diluted with 10 ml methanol and further suitably diluted with the mobile phase, filtered through a membrane filter and CUR quantified in triplicate by HPLC. The RP HPLC method was validated for linearity, accuracy, and precision.

#### 2.5.3 Stability

Preconcentrate was evaluated for stability as per International Conference on Harmonization (ICH) guidelines. Preconcentrate was packed in amber colored glass vials flushed with nitrogen and stored at 30 ± 2 °C/65 ± 5% RH and 40 ± 2 °C/75 ± 5% RH. At prescribed time intervals, preconcentrate was evaluated for drug content. At the same time intervals, ISCurNP was generated from the preconcentrate as described in 2.3.2 and evaluated for particle size and entrapment efficiency as described below.

### 2.6 Evaluation of ISCurNP

The preconcentrate was diluted with aqueous medium to generate ISCurNP which were evaluated as below:

#### 2.6.1 Particle size and zeta potential

Particle size (PS), polydispersity index (PDI) and zeta potential measurements of ISCurNP were evaluated on Nano Brook 90 Plus PALS (Brookhaven Instruments, NY, USA). Size distribution was determined by dynamic light scattering (DLS) at 25 °C. Zeta potential measurements were carried out using phase analytical light scattering (PALS). ISCurNP was suitably diluted with filtered distilled water and measurements were recorded in triplicate.

#### 2.6.2 Entrapment efficiency

The ISCurNP generated was centrifuged at 16,350 rcf for 20 min at 20 °C. Aliquot (1 ml) was carefully withdrawn from the supernatant, diluted to 10 ml with methanol and CUR concentration was estimated by UV spectrophotometry at λ_max_ 425 nm. EE was calculated using the equation below:

$$\%\mathrm{EE}=\left[\left({\mathrm{CUR}}_{\text{initial}}–{\mathrm{CUR}}_{\text{supernatant}}\right)/{\mathrm{CUR}}_{\text{initial}}\right]x\;100$$

#### 2.6.3 Particle imaging

##### Scanning electron microscopy

For scanning electron microscopy (SEM) analysis, a drop of ISCurNP was placed on a piece of aluminium foil mounted on carbon tube and air dried. Prior to analysis, sample was sputtered with platinum using an auto fine coater. Samples were analyzed using FEI Quanta 200 SEM with EDS.

##### Transmission electron microscopy

Transmission electron microscopy (TEM) analysis was performed using TECNAI 12 BT/FEI TEM at 120 kV. Briefly, a drop of ISCurNP was placed on carbon grid (Ted Pella, Inc., Redding) and air‐dried followed by negative staining with 2% uranyl acetate.

Following size and entrapment evaluation, ISCurNP dispersion was centrifuged, the pellet washed and redispersed in filtered distilled water and lyophilized using LABCONCO freeze dryer (FreeZone 4.5). This freeze‐dried solid sample was utilized for Differential scanning calorimetry (DSC), powder X‐ray diffraction (XRD) and Fourier‐transform infrared spectroscopy (FTIR) evaluation.

#### 2.6.4 Differential scanning calorimetry

DSC was performed under nitrogen purge (20 ml/min) using Perkin Elmer Pyris 6 DSC instrument (PerkinElmer, Netherlands). Thermograms were recorded by sealing samples (5 mg) in aluminium pans followed by heating over the temperature range 40–300 °C at rate of 10 °C/min. Empty sealed aluminium pan served as reference.

#### 2.6.5 Powder X‐ray diffraction

X‐ray diffraction spectra were recorded on Bruker X‐ray diffractometer under ambient conditions. Instrument was operated at 40 kV voltage and 40 mA current at 2θ/min scanning speed, scanning angles from 5 to 70^°^ (2θ) with a 0.2 step size.

#### 2.6.6 Fourier‐transform infrared spectroscopy

Samples were prepared in the form of KBr pellets and scanned from 3800 to 400 cm^−1^ using FTIR spectrophotometer (Perkin‐Elmer, Model Spectrum Two). A scan with blank KBr pellet eliminated background interference.

#### 2.6.7 Effect of dilution volume

Preconcentrate (0.75 ml) was maintained constant while the aqueous dilution volume was varied from 5–250 ml. The ISCurNP generated was evaluated for PS and EE.

### 2.7 In vitro release

Release of CUR from NPs was evaluated by a modified dialysis method. ISCurNP dispersion (5 ml) and free CUR suspended in 1% sodium lauryl sulfate (SLS) solution (5 ml) equivalent to 5 mg CUR were loaded into activated dialysis bags (Himedia 12–14 kDa molecular weight cut‐off). The dialysis bags were then introduced into the basket of USP dissolution apparatus I and immersed in 500 ml of dissolution medium maintained at 37 ± 0.5 °C. The rotation speed was 100 rpm. Physiological buffer solution pH 1.2 and pH 6.8 and artificial lysosomal fluid pH 4.5 (composition in Supporting Information Table S2), supplemented with 1% SLS were used as dissolution medium (500 ml). Aliquots (5 ml) were withdrawn at specific time intervals and estimated at λ_max_ of 425 nm by UV spectroscopy to quantify the amount of CUR released. Graphs of cumulative percent drug release versus time were plotted.

### 2.8 Cells

RAW 264.7 murine macrophage cell line was procured from American Type Culture Collection and grown in Dulbecco's modified eagle medium (DMEM) supplemented with 10% heat inactivated fetal bovine serum (FBS) and 1% penicillin/streptomycin at 37 °C in a humidified incubator with 5% CO_2_ until confluent. Cells were used from 3 to 12 passages.

#### 2.8.1 Sample preparation

Sterile preconcentrate was prepared by filtration through sterile 0.22 μm membrane filters under aseptic conditions. ISCurNP was generated by addition of 0.75 ml sterile preconcentrate to sterile distilled water to obtain CUR concentration 1 mg/ml. CUR stock was prepared by dissolving free CUR in cell culture grade dimethyl sulfoxide (DMSO) (5 mg/ml). All the concentrations were considered in μM corresponding to the total amount of CUR incorporated. Final dilutions were prepared in DMEM supplemented with 5% FBS. In situ generated Blank NP and DMSO served as controls.

#### 2.8.2 Stability of ISCurNP in culture medium

Aliquots of ISCurNP (equivalent to 100 μM) in DMEM, were placed in Eppendorf tubes and incubated at 37 °C and PS was recorded at 0, 3, 5, 24, 48, and 72 hr, using the Nano Brook 90 Plus Zeta instrument.

### 2.9 Cell viability assay

Cell viability was assessed by MTT (3‐[4,5‐Dimethylthiazol‐2‐yl]‐2,5‐Diphenyltetrazolium Bromide) assay. Cells (10,000 per well) were plated in sterile flat bottom 96‐well plates. Test samples were introduced, diluted by serial dilution technique and plates were placed in a CO_2_ incubator at 37 °C for 48 hr. Distilled water served as the positive control. Cells were washed with DMEM and incubated with 100 μl MTT (5 mg/ml) for 4 hr. Subsequently, 100 μl of 20% sodium dodecyl sulphate (SDS) was added to lyse the cells and solubilize the formazan crystals. Plate was read at 570 nm using plate reader (Biotek Multimode Reader) and results were normalized against untreated control. Percent cell viability was calculated using the following equation:

% cell viability = (Absorbance_sample_ /Absorbance_control_) x 100.

### 2.10 Macrophage uptake

Uptake of ISCurNP in the macrophages was evaluated flow cytometry, confocal microscopy and reverse phase HPLC.

#### 2.10.1 Flow cytometry

Cells (1 x 10^6^ cells/ ml) were seeded in 24‐well plates and incubated with samples (10 and 20 μM) or media as untreated control upto 3 hr. At 1 and 3 hr the samples were pipetted out, the cells were rinsed thrice with phosphate buffered saline (PBS) and detached by trypsinization. The resulting cell suspension was centrifuged at 10,460 rcf, 4 °C for 5 min with intermittent PBS washings. Cells were resuspended in PBS and intracellular fluorescence was acquired by CyFlow1 Space flowcytometer (Sysmex). Cells were gated according to forward scatter versus side scatter. Data were analyzed using FlowJo software (Treestar).

#### 2.10.2 Confocal microscopy

Cells (5 x 10^5^ cells/ml) were grown on sterile coverslips contained in 6‐well plates and placed in CO_2_ incubator overnight to obtain final cell density of 1 x 10^6^ cells/ml. Samples (10 and 20 μM) were added to the wells and allowed to incubate upto 3 hr. At 1 and 3 hr cells were washed with PBS thrice and fixed with 4% paraformaldehyde for 30 min. Coverslips were mounted on glass slides using Prolong® Gold Antifade Reagent with DAPI and sealed with nail paint. Imaging was done by Zeiss LSM 510 Meta confocal microscope (Carl Zeiss Microscopy) equipped with 63X oil immersion lens. At least three fields per slide were considered.

For evaluation of the subcellular localization of ISCurNP prior to addition of the samples, cells were incubated with 1:5,000 Lyso Tracker™ Red (lysosome specific dye) for 5 hr and the same procedure followed as above. Imaging was carried out at 24 hr.

#### 2.10.3 RP HPLC

##### HPLC method for estimation of CUR in macrophage cells

A suspension of RAW cells at a cell density 1 × 10^6^ cells/ml in DMEM containing 10% fetal calf serum was prepared. SDS in PBS (0.5%) was added in 1:1 ratio to enable cell lysis. The dispersion was centrifuged and the supernatant served as blank. To determine CUR recovery from macrophage cells, free CUR solution 100 μl (25 ng–2 μg/ml) was spiked to above supernatant (200 μl). Methanol (100 μl) was added to precipitate protein and the dispersion vortexed for 2 min and centrifuged at 10,460 rcf, 20 min at 20 °C. RP HPLC method developed in Section 2.5.2 was utilized to analyze the recovery of CUR from blank cell suspension.

##### Macrophage uptake of ISCurNP

For estimation of CUR uptake in macrophages, cells (1 x 10^6^ cells/ml) were seeded in 12‐well plates and incubated with samples (20 μM) upto 3 hr. At particular time point, media containing samples was removed and analyzed for uninternalized CUR content by centrifuging at 10,460 rcf, 4 °C for 5 min. The supernatant was treated with methanol in 1:1 ratio to precipitate proteins, centrifuged and analyzed. To assess the internalized NPs, cell pellet was lysed by addition of 0.5% SDS in PBS. Methanol was added to the lysed cells in 1:1 ratio to precipitate proteins, samples centrifuged and analyzed.

### 2.11 Statistical analysis

All experimental results are expressed as mean ± standard deviation of minimum three independent measurements. Student's *t*‐test was applied for statistical analysis and *p* < .05 has been considered as statistically significant.

## 3 RESULTS AND DISCUSSION

### 3.1 Fabrication and optimization of nanoparticles

A prerequisite for in situ nano drug delivery system is a pharmaceutically acceptable water miscible solvent that can solubilize the drug and excipients. While CUR exhibited limited solubility in most solvents, maximum and high solubility was observed in DMA (Supporting Information Table S3). Soluplus® and PLGA revealed adequate solubility of >100 mg/ml in DMA. Hence, DMA was selected as the vehicle for development of ISCurNP.

ISCurNP revealed rapid aggregation in the absence of a stabilizer necessitating addition of stabilizer(s) to ensure a uniform and stable nanodispersion. Kolliphor P188 (polyoxyethylene‐polyoxypropylene block‐co‐polymer) is reported as a stabilizer for nanosystems.^23^, ^24^, ^25^ Soluplus® (polyvinyl caprolactam‐polyvinyl acetate‐polyethyleneglycol graft copolymer) is reported to effectively stabilize wet milled fenofibrate NPs by decreasing the interfacial tension.^26^ Both these stabilizers were evaluated. Kolliphor P188 could not mitigate NP aggregation even at a concentration of 1%. Hence, Soluplus® was selected. Preliminary screening using OVAT approach revealed a marked influence on PS and EE when Soluplus® and DMA were varied. While varying PLGA concentration revealed no significant change on PS, moderate influence on EE was seen (Supporting Information Figure S1).

Design of experiment‐based approaches can assess single variables as well as interactions between different variables, enabling optimization with fewer experiments, resulting in improved efficiency.^27^, ^28^, ^29^ Two‐level designs like full or fractional factorial design provide restricted statistical outputs. Among the response surface designs, which are based on more than two‐factor levels, the most efficient and widely used design is the three‐level Box–Behnken design.^30^, ^31^, ^32^ In this design, a midpoint of each side of a multidimensional cube is selected and set of points around these are chosen whereby extreme values are avoided. Furthermore, interactions between the independent variables are also accounted for.

The desired levels of the dependent variables were average PS (Y1): 200–400 nm, %EE (Y2): ≥85%. Box–Behnken design generated a matrix of 15 experimental runs. The PS and %EE of different batches is depicted in Supporting Information Table S4. Regression analysis was carried out and *p* < .05 was deemed statistically significant. Significant model fits for PS and %EE with *R* ^2^ values of 0.95 and 0.87 respectively were obtained. anova test revealed *p* < .05 for all factors suggesting reliable predicted results. The following regression equations were generated:

$$Y_{1}=49.5+10.89\;X_{1}+22\;X_{3}+12.50\;X_{2}-0.341\;{X_{1}}^{*}X_{1}-130.5\;{X_{3}}^{*}X_{3}-0.1595\;{X_{2}}^{*}X_{2}+1.28\;{X_{1}}^{*}X_{3}-0.0527\;{X_{1}}^{*}X_{2}-3.83\;{X_{3}}^{*}X_{2}$$

$$Y_{2}=79.7-0.07\;X_{1}+84.6\;X_{3}-0.741\;X_{2}-0.0881\;{X_{1}}^{*}X_{1}-120.8\;{X_{3}}^{*}X_{3}-0.0128\;{X_{2}}^{*}X_{2}+1.65\;{X_{1}}^{*}X_{3}+0.0477\;{X_{1}}^{*}X_{2}+1.507\;{X_{3}}^{*}X_{2}$$

**Figure 1:**
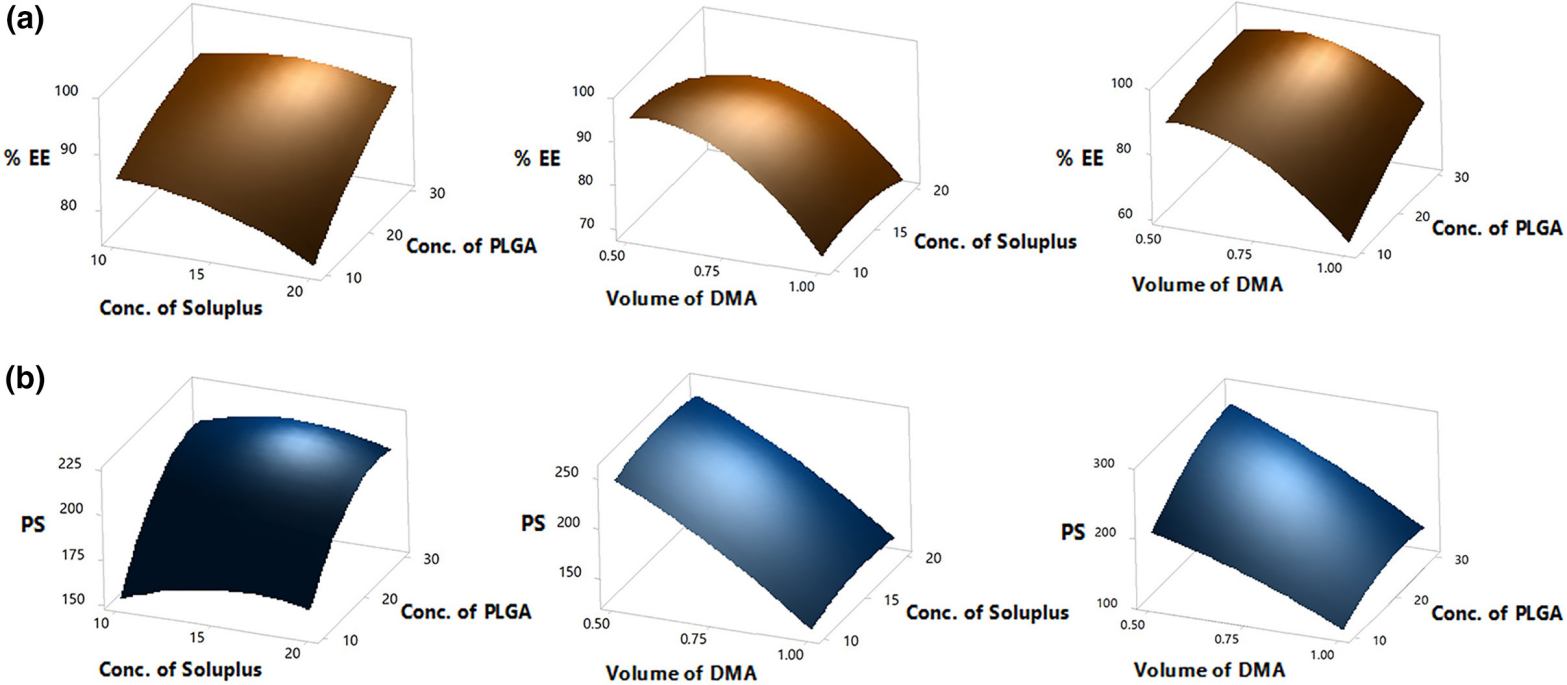
Surface plots of effect of change in concentration of Soluplus®, concentration of PLGA and volume of DMA on (a) Percent entrapment efficiency and (b) average particle size (*n* = 3, mean ± SD)

The surface plots (Figure 1) depicted a decrease in %EE with an increase in Soluplus® concentration. The decrease in %EE with increasing Soluplus® concentration was attributed to its extremely low critical micellar concentration (0.00076%), that resulted in enhanced CUR solubilization. However, there was no significant effect (*p* > .05) of varying Soluplus® concentration on PS. Increasing the volume of DMA revealed a decrease in the %EE and PS. This was due to enhanced solubilization of CUR in DMA. In contrast, PS increased with an increase in PLGA concentration. Increasing polymer concentration results in increased polymer–polymer interaction and viscosity, resulting in slower solvent diffusion into the nonsolvent or aqueous phase thereby forming larger NPs.^33^

Three test compositions ISCurNP1, ISCurNP2, and ISCurNP3 were generated using the design and the dependent variables predicted (Table 1). The actual data generated for these compositions closely matched the predicted data (*p* > .05), indicating robustness of the mathematical model, thus validating the experimental design. All the three batches revealed desirable responses. Nevertheless, ISCurNP1 and ISCurNP3 exhibited faster particle aggregation suggesting lower physical stability. This is attributed to the lower concentration of the stabilizer Soluplus® in ISCurNP1 and the smaller volume of DMA in ISCurNP3. ISCurNP2 was considered as the optimized batch and selected for further studies.

**Table 1:** Predicted and observed values for NPs (*n* = 3, mean ± SD)

				PS (nm)		%EE	
Batch	Concentration of Soluplus® (mg)	Concentration of PLGA (mg)	Volume of DMA (ml)	Predicted	Observed	Predicted	Observed
ISCurNP1	12.5	20	0.7	214.3	218.8 ± 12.17	92.62	91.96 ± 0.85
ISCurNP2	15	20	0.75	206.48	208.25 ± 7.55	89.88	90.16 ± 1.17
ISCurNP3	15	15	0.5	235.08	232.09 ± 12.46	91.37	90.8 ± 1.03

#### 3.1.1 Evaluation of preconcentrate

Selected preconcentrate (ISCurNP2) was found to be clear when visualized against light and dark background. CUR content in the preconcentrate was 99.5 ± 0.85%. At the end of 6 months, CUR content of >95% was observed following exposure to 40 ± 2 °C/75 ± 5% RH. Long‐term stability data indicated >95% CUR content at end of 12 months following exposure to 30 ± 2 °C/65 ± 5% RH. Furthermore, no significant change in PS and %EE was observed on generation of ISCurNP. This confirmed good stability and a predicted shelf life of minimum 2 years as per ICH guidelines.

#### 3.1.2 Evaluation of ISCurNP

**Figure 2:**
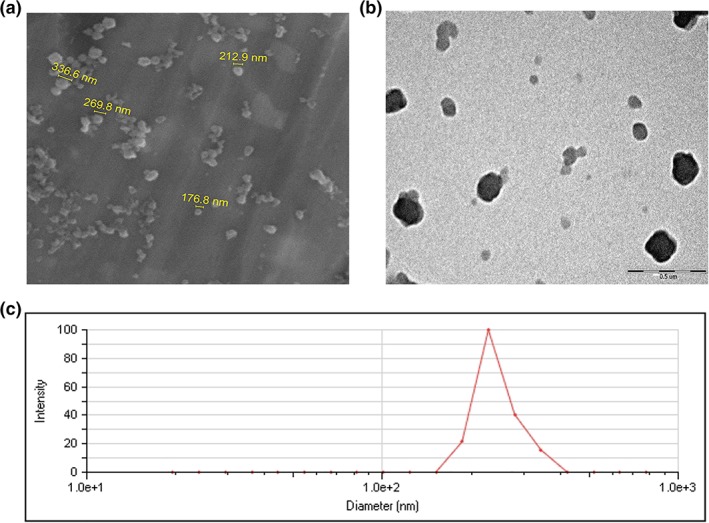
Particle imaging: (a) SEM micrograph, (b) TEM micrograph, and (c) particle size distribution of ISCurNP

ISCurNP revealed PS, PDI, and %EE of 208.25 ± 7.55 nm, 0.165 ± 0.019, and 90.16 ± 1.17% respectively. A zeta potential of −26 ± 2.55 mV indicated good colloidal stability. SEM (Figure 2a) and TEM (Figure 2b) revealed nearly spherical morphology with relatively smooth surface. The size obtained by SEM and TEM correlated with DLS values. PS distribution is depicted in Figure 2c.

Amorphization of highly insoluble drugs like CUR is one strategy to enhance bioavailability. Absence of sharp endothermic peak of CUR at 183 °C, in the DSC thermogram indicated conversion of crystalline CUR to amorphous form (Supporting Information Figure S2). The disappearance of the peaks characteristic of crystalline CUR in the XRD spectra also confirmed amorphization of CUR (Supporting Information Figure S3).

FTIR spectra of ISCurNP (Supporting Information Figure S4) depicted presence of C=O stretch at 1,625 and 1,762 cm^−1^ corresponding to carbonyl groups in CUR and PLGA respectively. Additionally, C‐H and O‐H stretching was observed at 2,884 and 3,460 cm^−1^, respectively. Presence of bands in ISCurNP corresponding to bands in CUR and PLGA confirmed incorporation of CUR in the NPs.

NPs, due to their small size, large surface area, and surface properties are prone to aggregation. Although there was good stability up to 24 hr as indicated by PS, small aggregates were seen after 6 hr. Nevertheless, these aggregates could be readily redispersed by mild shaking and revealed good stability when monitored on PS analyzer, suggesting formation of floccules. As ISCurNP is designed for immediate administration, even 6 hr stability was considered more than adequate.

During the optimization study, dilution volume was maintained constant at 10 ml. When injected, dilution would be carried out by trained health professionals and hence a dilution volume may be prescribed. However, when administered orally the dilution would be carried out by untrained users. To understand the ruggedness under such conditions of use, we evaluated the effect of varying the dilution volume on PS and EE (Supporting Information Figure S5). ISCurNP revealed no significant change in PS and EE when the dilution volume was varied from 10 ml (two teaspoons) to 100 ml (about half a glassful; *p* > .05). At 5 ml faster aggregation was seen. However, a 3 hr stability was observed which was considered satisfactory. At >100 ml, a significant decrease in PS was observed (*p* < .05). Although considered rugged to dilution, as a consideration in translation a measuring cup of about 50–75 ml may be provided to ensure dilution volume within the desirable range.

##### In vitro release

**Figure 3:**
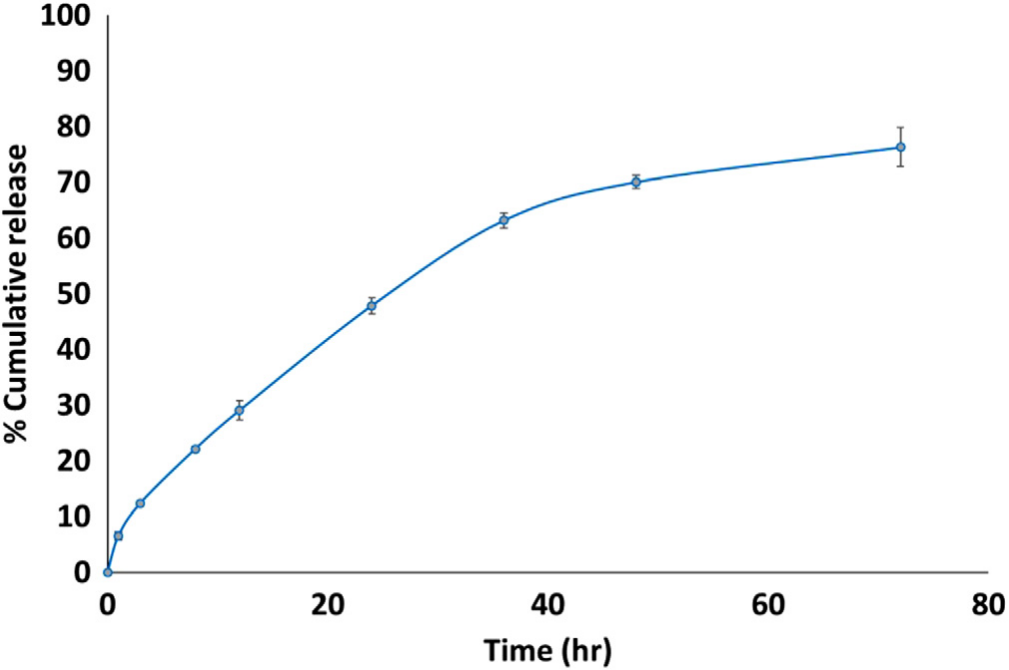
In vitro release of CUR from ISCurNP in artificial lysosomal fluid pH 4.5 (*n* = 3, mean ± SD)

In vitro release of ISCurNP was studied at pH 1.2 and pH 6.8 to mimic the pH in the stomach and intestine, respectively. At pH 1.2, no release was observed upto 2 hr from ISCurNP suggesting that the NPs would remain intact in the stomach. However, at pH 6.8 sustained release of CUR from ISCurNP was observed upto 24 hr with maximum 46.6% release while free CUR demonstrated of 28.09% release (Supporting Information Figure S6). We have previously observed that hydrophilic NPs show gastric tropism^34^ while hydrophobic NPs favor intestinal accumulation^35^. Such intestinal accumulation could favor high uptake of intact NPs through M cells of Peyer's patches followed by translocation through the lymph into systemic circulation.^29^ In an analogous manner, we hypothesize that ISCurNP being hydrophobic could exhibit intact NP uptake through Peyer's patches, and translocate through lymph to systemic circulation to favor macrophage uptake. Release was also evaluated in artificial lysosomal fluid pH 4.5 to simulate the environment in the macrophages. NPs revealed 76.29 ± 3.5% release of CUR from ISCurNP at 72 hr, suggesting slow but near complete release (Figure 3). Model fitting revealed CUR release from ISCurNP followed Higuchi model at both pH 4.5 and 6.8 (Supporting Information Table S5).

##### Stability in culture medium

Biological media components could adsorb onto NPs to alter their surface characteristics and lead to aggregation. Such aggregated NPs can alter the cellular uptake, cytotoxicity or even the in vivo fate.^36^ Physical stability (size) of NPs in cell culture medium was evaluated to ensure that they do not aggregate and remain discrete when evaluated in cell lines. No significant change in PS was seen upto 48 hr (*p* > .05). At 72 hr, although the size increased, the average size of <400 nm was considered acceptable (Supporting Information Figure S7).

##### Cell viability assay

**Figure 4:**
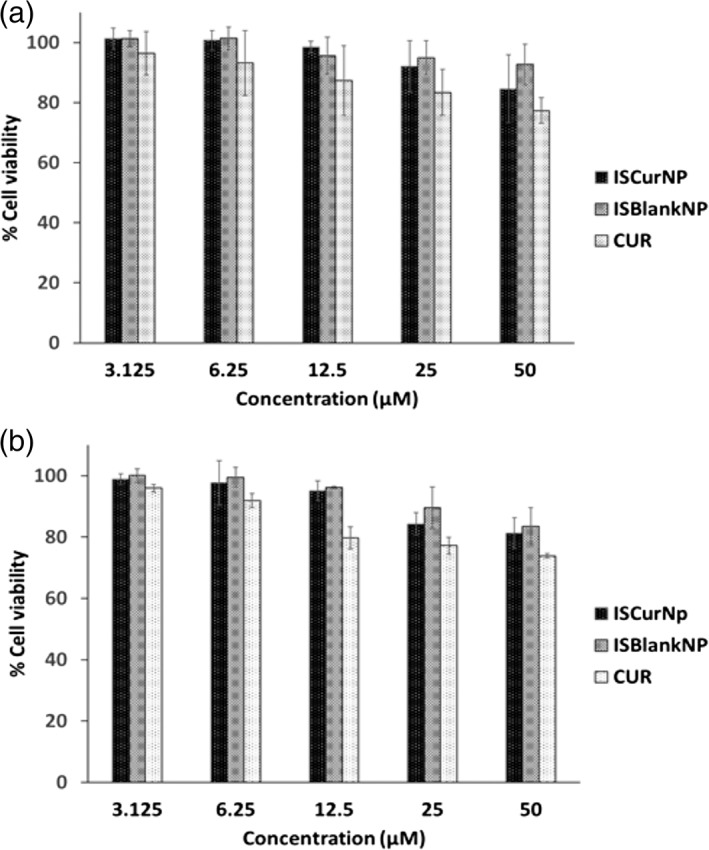
Percent cell viability of RAW 264.7 cells by MTT assay: (a) 24 hr post treatment (b) 48 hr post treatment. (n = 3, mean ± SD)

MTT assay is widely used for assessing cytotoxicity in cell lines. MTT (3‐[4,5‐Dimethylthiazol‐2‐yl]‐2,5‐Diphenyltetrazolium Bromide), a water soluble yellow colored tetrazolium dye readily enters the cells and is converted into purple formazan crystals only in viable cells in presence of mitochondrial dehydrogenase enzyme which is evaluated by colorimetric assay.^37^ The ISCurNP and ISBlankNP revealed minimal cytotoxicity (Figure 4). Even at 48 hr, >80% cell viability was observed suggesting suitability for further studies.

### 3.2 Macrophage uptake

Macrophages rapidly engulf and internalize all foreign invasions including organisms and particles by phagocytosis.^38^ Typically, hydrophobic particles are taken up by this pathway.^39^, ^40^ Phagocytosis results in trapping of the foreign invasion in a phagosome which coalesce with the lysosomes present in the macrophages to form phago‐lysosomes. The harsh phagolysosomal environment which has a low pH and lysosomal enzymes results in degradation/killing of the internalized organism. However, organisms that have mastered the art of phagosomal survival can competently arrest early phagosome maturation and hinder phagolysosome fusion.^41^ The phagosome provides a safe sanctuary for such organisms. Given time, some of these organisms can also generate a protective resistant shield as in case of *Mycobacterium tuberculosis*.^42^ Elimination of such intracellular infections poses significant challenges dictating the need for intracellular delivery of the drug at therapeutic concentration.^18^, ^43^, ^44^ Nanocarrier‐based delivery is an effective strategy for high intracellular delivery, nevertheless yet another challenge is to ensure that the drug from the nanocarrier is released out of the phagolysosome into the cytoplasmic compartment which harbors the organisms. In the present study, we monitored ISCurNP uptake and also quantified CUR concentration in the RAW 264.7 macrophage cell line.

**Figure 5:**
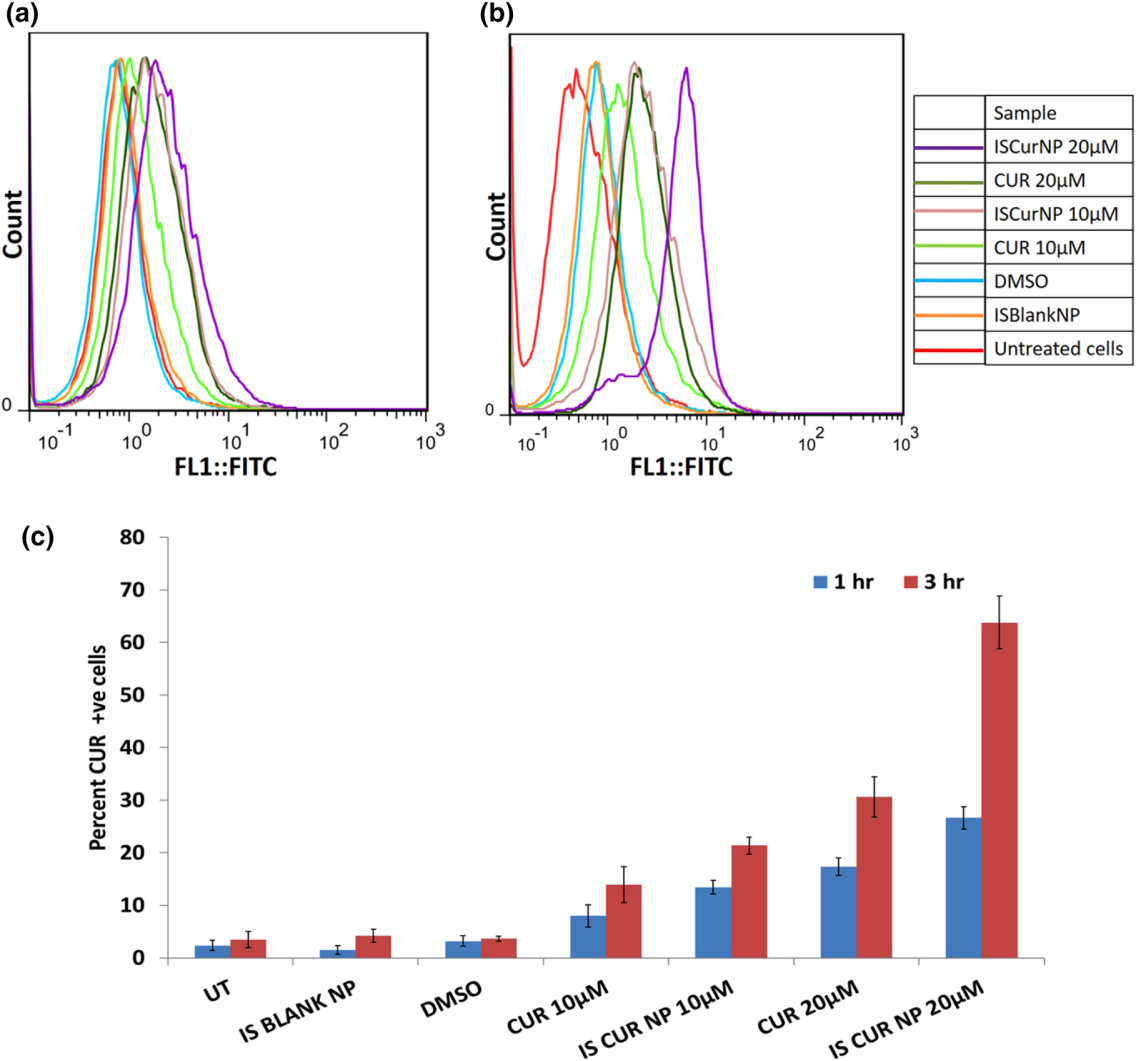
Flow cytometry histogram depicting uptake at (a) 1 hr, (b), and 3 hr, (c) graphical representation of uptake at 1 and 3 hr (*n* = 3, mean ± SD)

Flow cytometry analysis relied on fluorescence emission of CUR. The concentration and time dependent uptake of CUR is depicted in Figure 5. Both free CUR and ISCurNP revealed time dependent increase in uptake at both concentrations (*p* < .05). Significant uptake at 1 hr confirmed rapid internalization of ISCurNP. Further, uptake exhibited by ISCurNP was significantly higher than free CUR (*p* < .05). At 3 hr, while increase in CUR concentration from 10 to 20 μM revealed approximate twofold increase, this increase was approximately threefold when the ISCurNP concentration was similarly increased.

**Figure 6:**
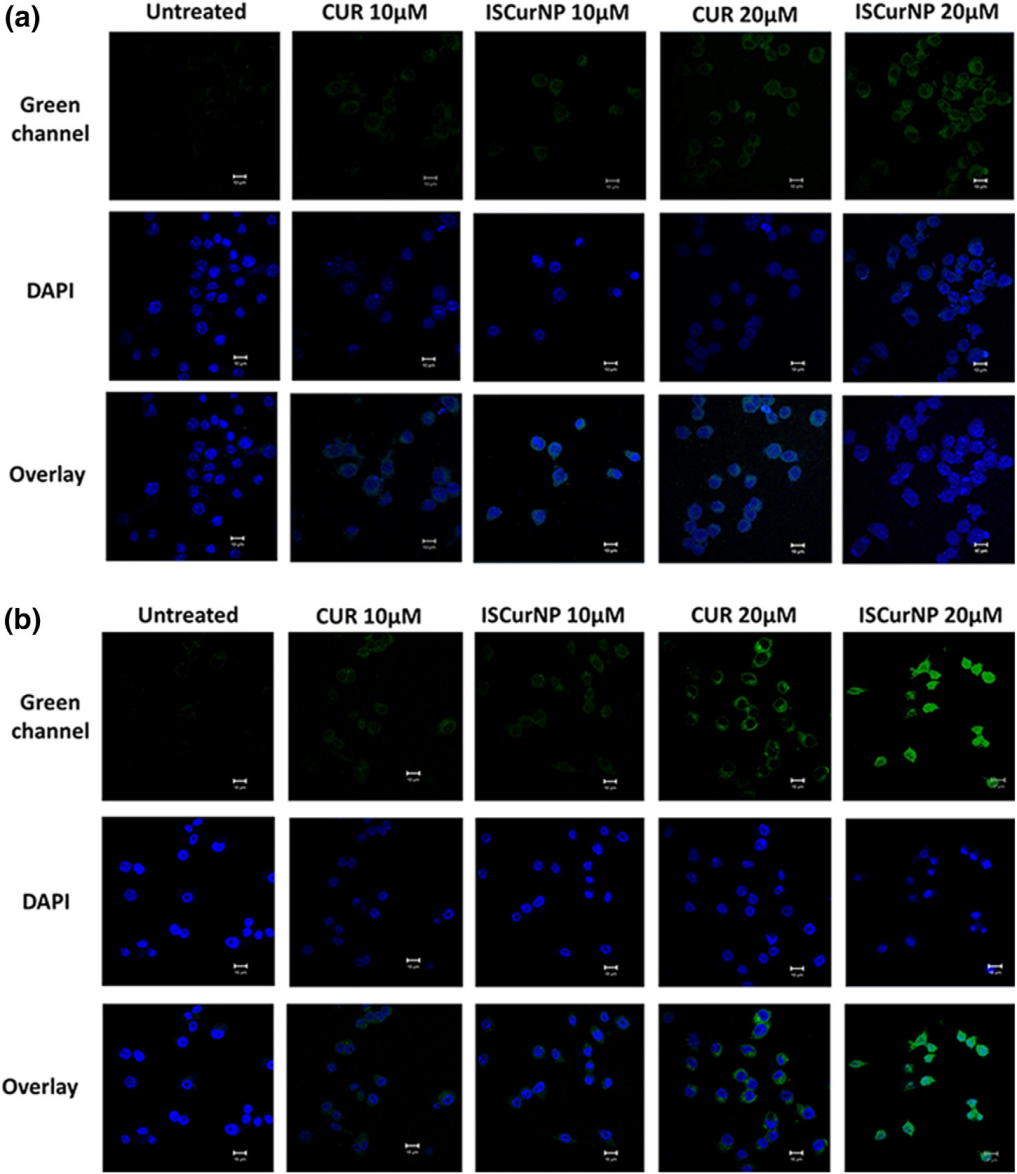
Confocal images depicting uptake of CUR and ISCurNP at (a) 1 hr and (b) 3 hr in RAW 264.7 cell line. Green fluorescence arises from curcumin whereas blue fluorescence arises from DAPI stained nuclei. Scale bar:10 μm

Uptake of ISCurNP by macrophages was also monitored by confocal microscopy to understand intracellular trafficking (Figure 6). As seen with flow cytometry, both concentration and time‐dependent increase in uptake was observed. Maximum and significant uptake was seen at 3 hr with the ISCurNP. Although no significant difference in uptake was observed at 10 and 20 μM between CUR and ISCurNP at 1 hr, ISCurNP revealed a significantly enhanced fluorescence at 3 hr, suggesting intracellular uptake.

**Figure 7:**
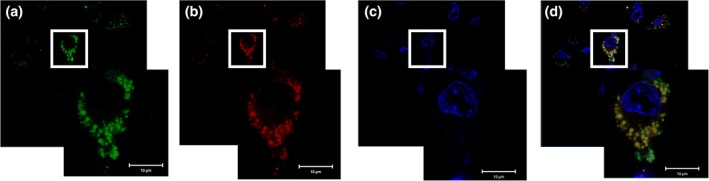
Co‐localization of ISCurNP with phago‐lysosomal compartments in RAW 264.7 cell line: (a) ISCurNP (green), (b) acidic compartments stained with Lysotracker™ red (red), (c) DAPI stained nuclei (blue), and (d) overlay of ISCurNP, Lysotracker™ red and DAPI (yellow). Scale bar:10 μm

Subcellular localization of NPs has been a subject of dispute.^45^, ^46^, ^47^ While rapid cytoplasmic escape of phagocytosised NPs has been demonstrated in few studies, others claim an extended residence in the phago‐lysosomes. Kalluru et al. observed co‐localization of PLGA NPs with Lyso Tracker™ Red, DQRed BSA and LAMP2 for at least a week in the phago‐lysosomal compartment. The electron microscopy results confirmed 93% co‐localization with the lysosomal markers.^45^ We monitored the subcellular localization of NPs using Lyso Tracker™ Red and DAPI which localize in the lysosomes and nucleus respectively (Figure 7). Our study was in concurrence with results of Kalluru et al. wherein ISCurNP colocalised (yellow color) with LysoTracker™ Red confirming extensive retention in the lysosomes at 24 hr. Nevertheless, it is earlier demonstrated that PLGA NPs degraded slowly by hydrolysis in the aqueous lysosomal media over days or weeks to slowly release the drug that could then diffuse in the cytoplasm and perhaps later out of the cells.^45^ For drugs that are stable in lysosomal environment, this could provide a strategy for drug delivery to the cytoplasmic compartment desirable for efficacious treatment of many intracellular infections wherein the organism localizes and harbors in phagosomes. Slow release from ISCurNP over 3 days in simulated lysosomal media suggested high probability of ISCurNP being delivered into the cytoplasmic compartment.

As a further step, although we could not monitor cytoplasmic concentration of CUR, we monitored CUR uptake from ISCurNP in the macrophages by RP HPLC. The RP HPLC method was found to be accurate, precise with linearity range of 25 ng–2 μg/ml. Recovery of CUR from cells was found to be very good at 96.78 ± 2.91%. CUR was monitored in the medium in which the cells were exposed and also in the cells after lysis. A mass balance of >95% indicated appropriateness of the method. The uptake of CUR and ISCurNP at 1 hr was 15.99 ± 0.31% and 52.34 ± 8.67%, respectively. At 3 hr, enhanced uptake was seen CUR (29.22 ± 2.32%) and ISCurNP (89.24 ± 1.64%). The threefold enhancement in uptake observed for ISCurNP compared to CUR indicated phagocytosis mediated augmented intracellular uptake. Such uptake suggests targeted delivery and improved therapeutic efficacy of ISCurNP in intracellular infections.

## 4 CONCLUSION

In situ nanoprecipitation presents a facile approach for development of polymeric curcumin NPs. The low cytotoxicity and high uptake of ISCurNP in the RAW 264.7 cells confirms macrophage targeting and potential application in the therapy of intracellular infections. Based on technology that is simple and scalable, ISCurNP presents a targeted nanocarrier system with ease of translation from bench to clinic.

## CONFLICT OF INTEREST

The authors declare that there is no conflict of interest.

## Supporting information

Appendix S1: Supporting Information
