# Supplementary material for: Polymeric curcumin nanoparticles by a facile in situ method for macrophage targeted delivery
Source: Bioeng Transl Med. 2018 Nov 5;4(1):141–51. doi: 10.1002/btm2.10112 (PMC6336664; doi:10.1002/btm2.10112)
Supplement: Supplementary file 1 — Appendix S1: Supporting Information [file BTM2-4-141-s001.docx]

**Supplementary Tables**

**Table S1:** Factors and Levels considered for Box Behnken design

| Factors | Level | | |
| --- | --- | --- | --- |
|  | **Low (-1)** | **Middle (0)** | **High (+1)** |
| X1: Concentration of Soluplus^®^ (mg) | 10 | 15 | 20 |
| X2: Concentration of PLGA (mg) | 10 | 20 | 30 |
| X3: Volume of DMA (mL) | 0.5 | 0.75 | 1 |

**Table S2:** Composition of Artificial Lysosomal Fluid pH 4.5

| Chemical | g/L |
| --- | --- |
| Magnesium chloride | 0.0497 |
| Sodium chloride | 3.21 |
| Disodium phosphate | 0.071 |
| Sodium sulfate | 0.039 |
| Calcium chloride dihydrate | 0.128 |
| Sodium hydroxide | 6 |
| Citric acid | 20.8 |
| Glycine | 0.059 |
| Trisodium citrate | 0.077 |
| Sodium tartrate | 0.090 |
| Sodium lactate | 0.085 |
| Sodium pyruvate | 0.086 |

**Table S3:** Solubility of CUR in different solvents

| Solvent | Solubility in mg/mL |
| --- | --- |
| DMA | >500 mg/mL |
| Propylene glycol | 1.72±0.27 |
| Transcutol HP | 6.93±0.77 |
| PEG 300 | 9.15±1.27 |
| PEG 400 | 6.29±1.69 |

* Each value represents mean ± S.D. (n=3)

**Table S4:** Software generated compositions and recorded responses in Box Behnken design

| Run Order | Parameters | | | Responses | |
| --- | --- | --- | --- | --- | --- |
|  | **X1:** **Concentration of Soluplus^®^ (mg)** | **X2:** **Concentration of PLGA (mg)** | **X3:** **Volume of DMA (mL)** | **Y1:Particle size (nm)** | **Y2:Entrapment Efficiency (%)** |
| 1 | 15 | 20 | 0.75 | 203.65±15.04 | 87.5±2.51 |
| 2 | 10 | 30 | 0.75 | 212.08±12.86 | 94.63±1.37 |
| 3 | 20 | 20 | 0.5 | 262.63±14.6 | 91.5±0.47 |
| 4 | 10 | 20 | 0.5 | 247.06±9.36 | 93.86±2.66 |
| 5 | 20 | 30 | 0.75 | 199.47±5.57 | 87.12±1.96 |
| 6 | 15 | 10 | 1 | 115.87±3.74 | 61.9±2.69 |
| 7 | 20 | 10 | 0.75 | 157.87±6.48 | 73.41±3.1 |
| 8 | 10 | 10 | 0.75 | 159.29±8.29 | 90.56±1.3 |
| 9 | 15 | 30 | 1 | 147.63±3.27 | 83.83±4.55 |
| 10 | 15 | 10 | 0.5 | 197.97±0.31 | 85.8±3.2 |
| 11 | 15 | 30 | 0.5 | 268.05±11.21 | 92.98±0.44 |
| 12 | 10 | 20 | 1 | 113.8±4.64 | 67.28±7.89 |
| 13 | 20 | 20 | 1 | 135.75±3.35 | 71.78±3.56 |
| 14 | 15 | 20 | 0.75 | 200.87±9.73 | 90.7±1.09 |
| 15 | 15 | 20 | 0.75 | 200.34±21.76 | 92.3±0.64 |

**Table S5:** Model fitting for *in vitro* release

| Release media | R^2^ values | | | | Best fit model |
| --- | --- | --- | --- | --- | --- |
|  | **Zero order** | **First order** | **Higuchi** | **Korsemeyer Peppas** |  |
| Artificial lysosomal fluid pH 4.5 | 0.897 | 0.967 | **0.982** | 0.858 | Higuchi |
| Physiological buffer pH 6.8 | 0.9 | 0.964 | **0.97** | 0.94 | Higuchi |

**Supplementary Figures**


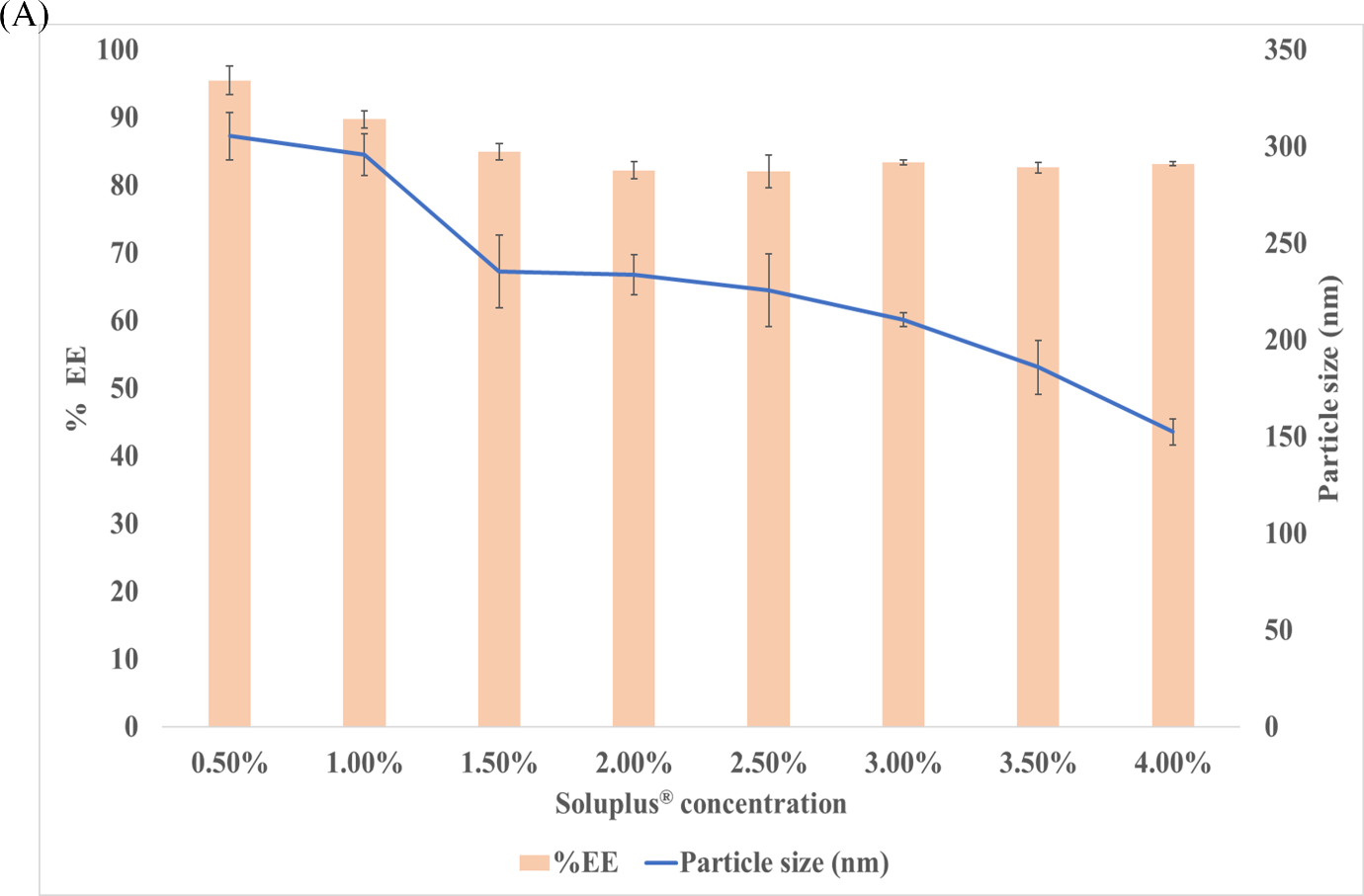


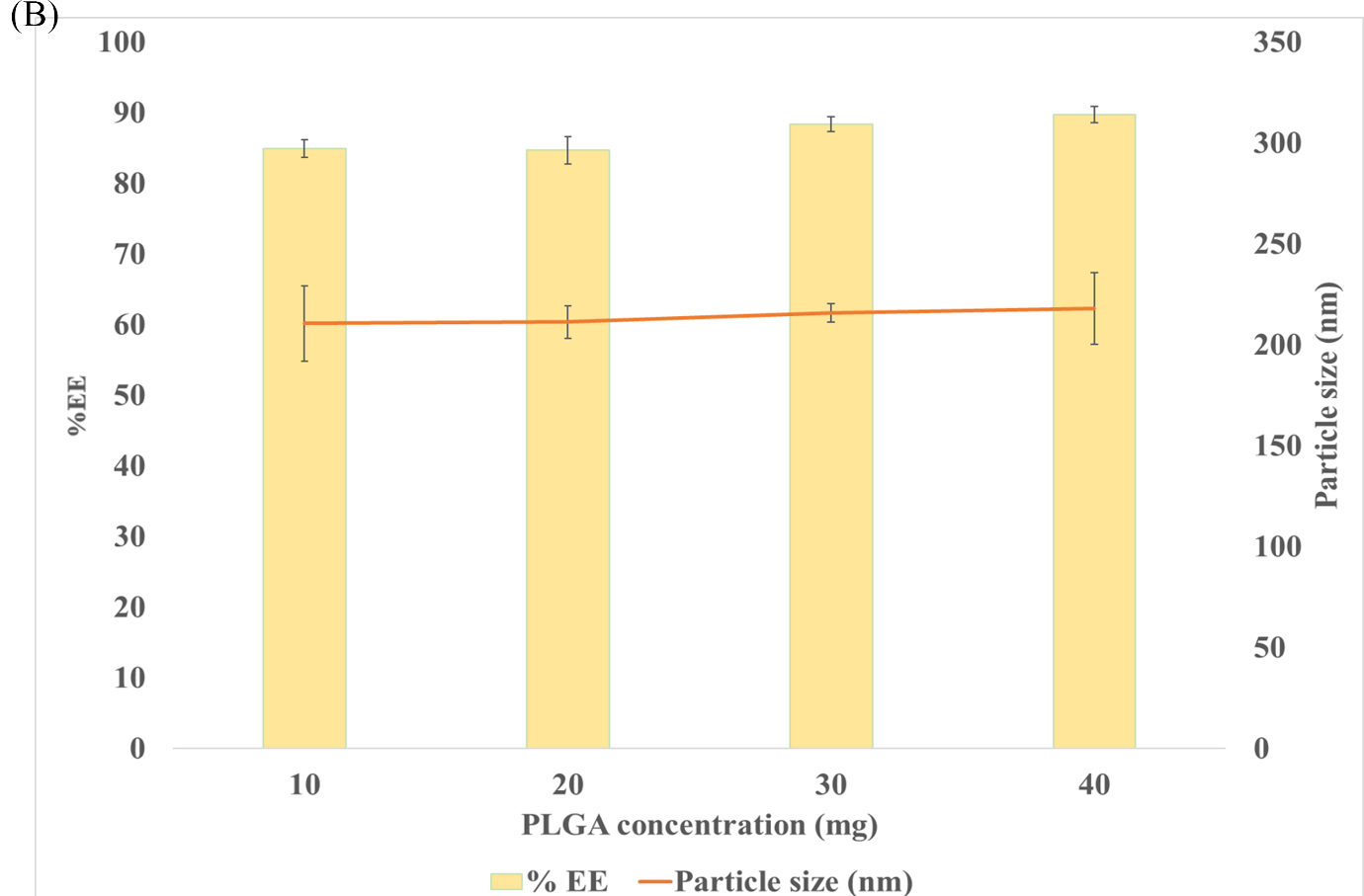


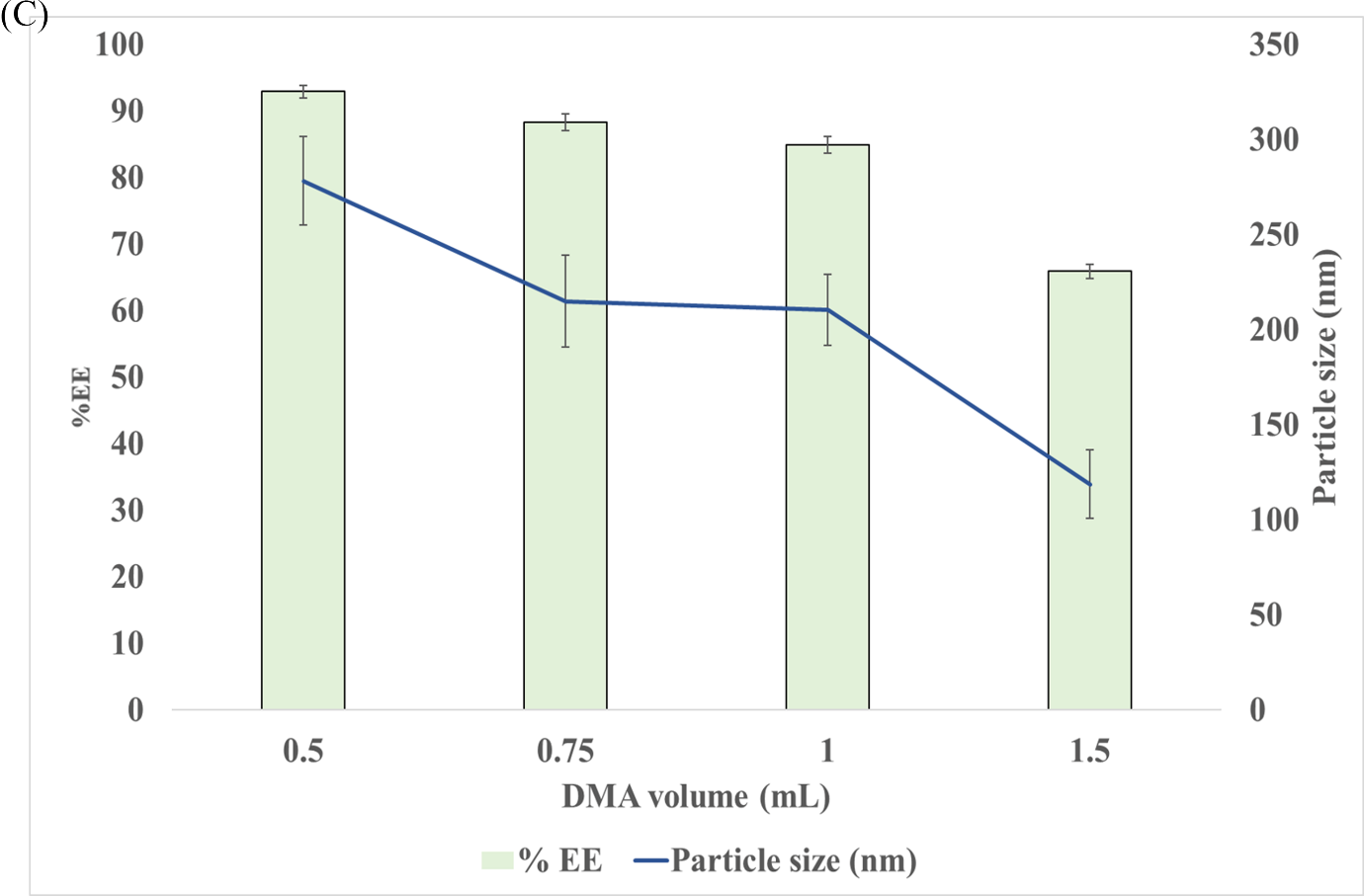


**Figure S1:** Preliminary screening by OVAT approach (A) Effect of Soluplus® concentration (B) Effect of PLGA concentration (C) Effect of change of DMA volume

**
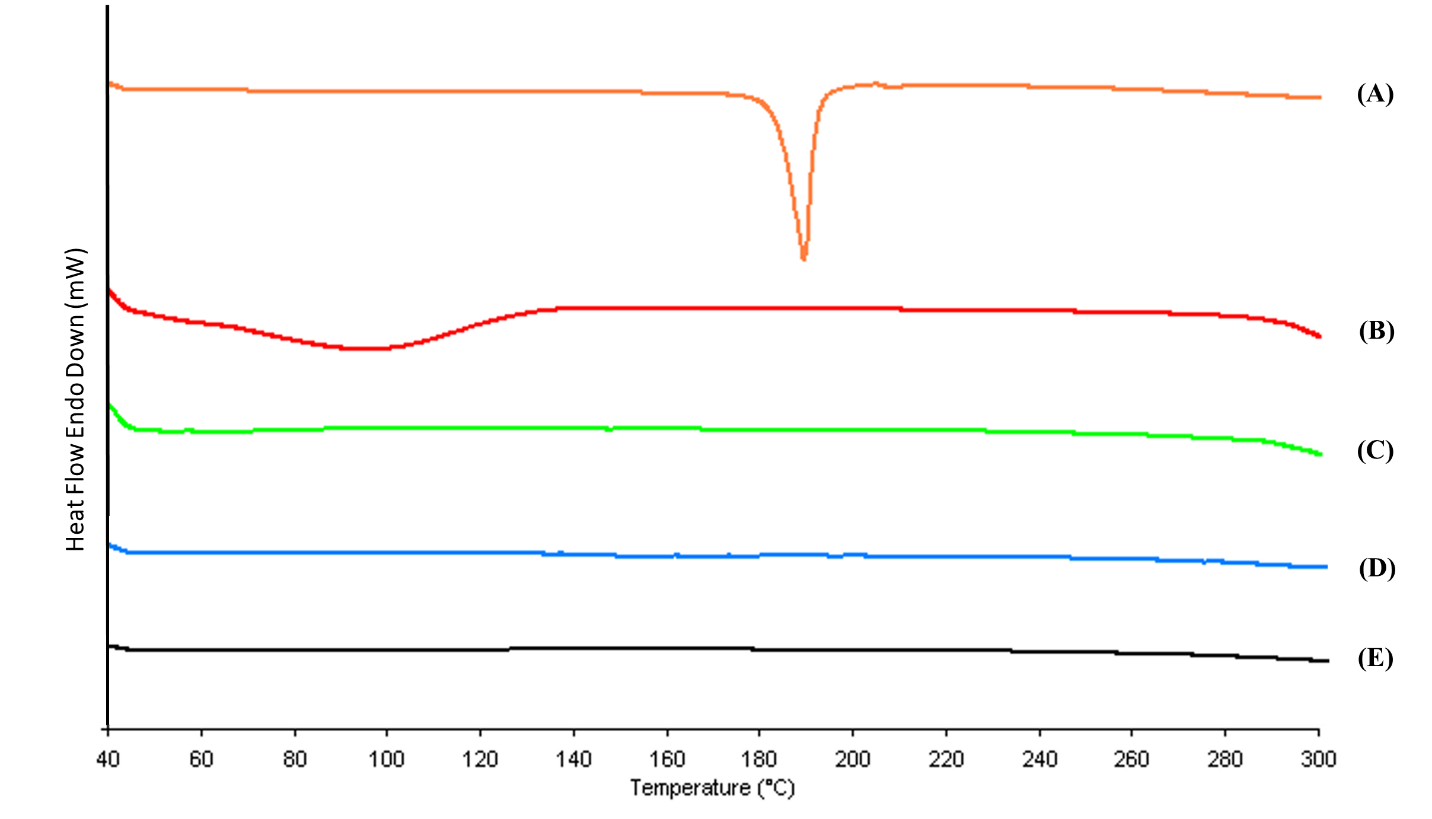
Figure S2**: DSC Thermograms of (A) CUR, (B) Soluplus, (C) PLGA, (D) ISBlankNP and (E) ISCurNP


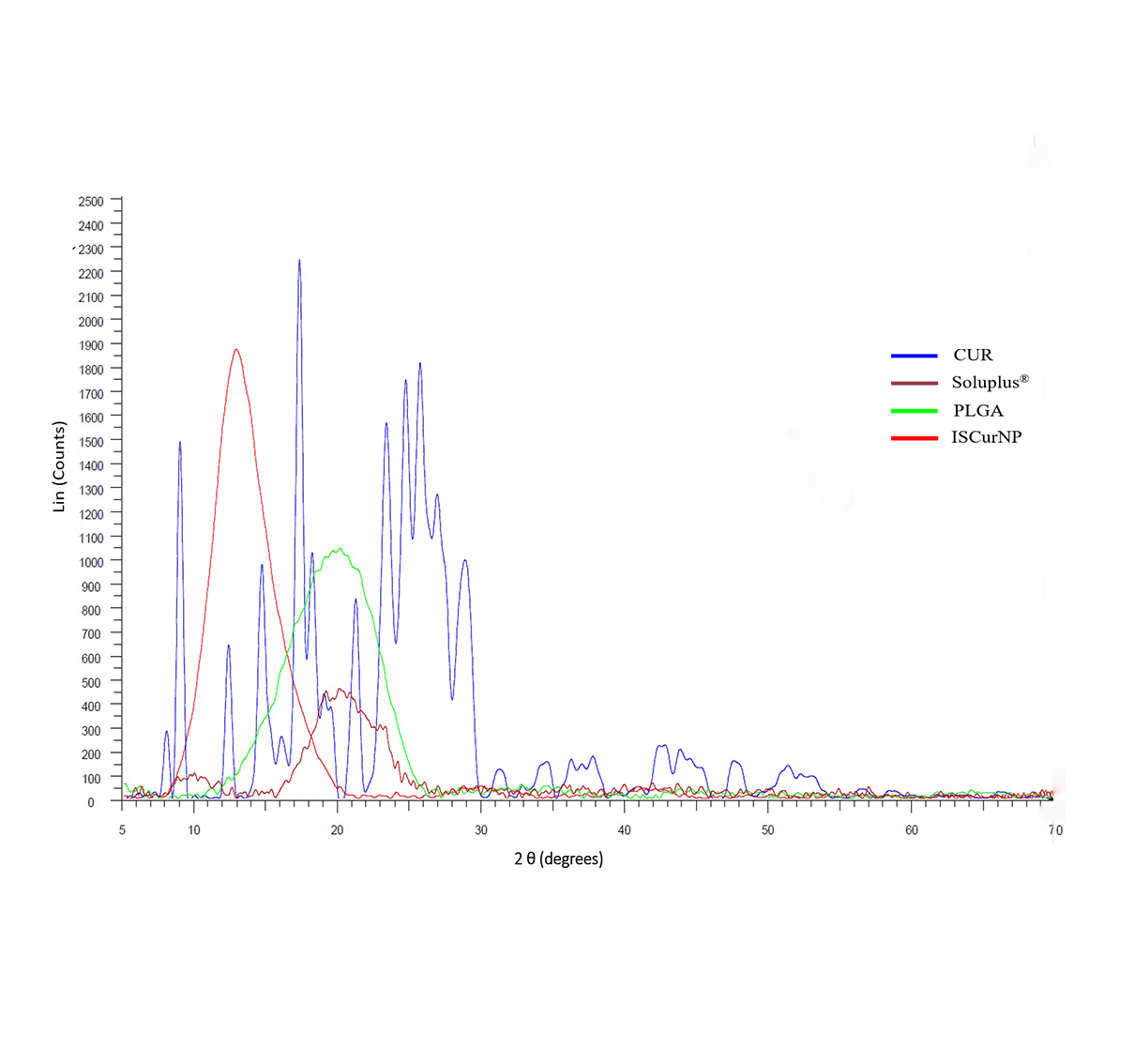
**Figure S3:** X-Ray diffraction patterns of CUR, Soluplus^®^, PLGA and ISCurNP


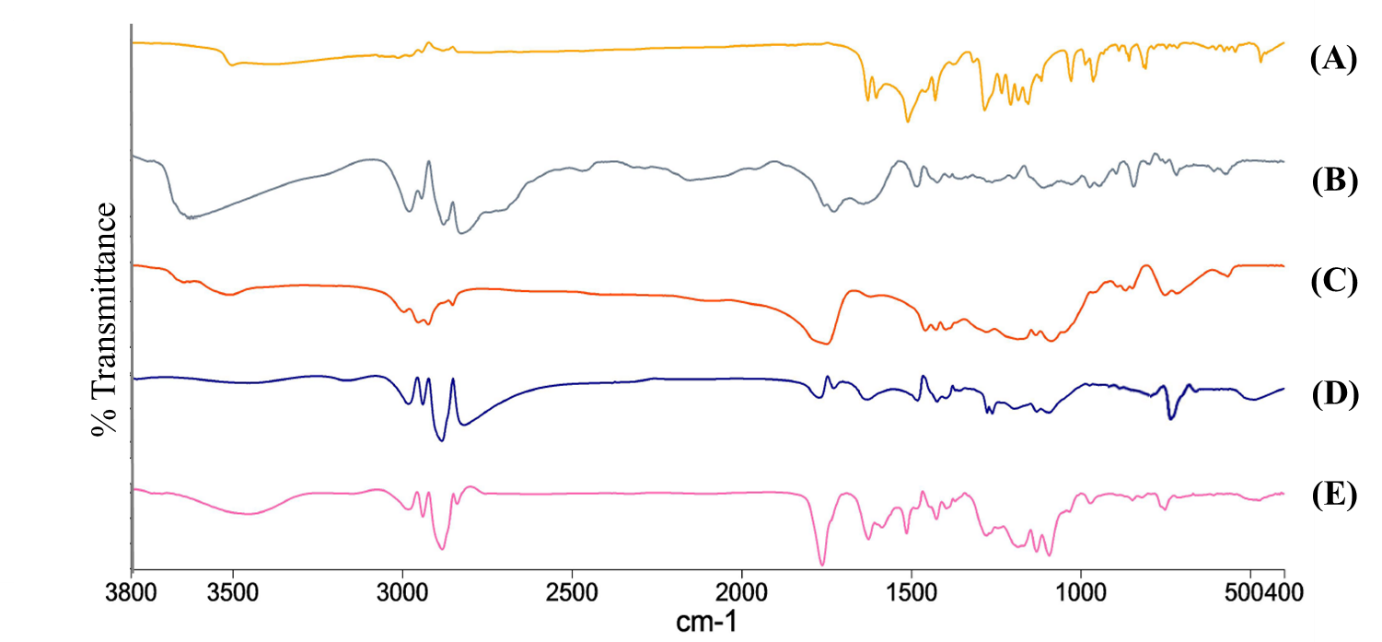


**Figure S4:** FTIR spectra of (A) CUR, (B) Soluplus^®^, (C) PLGA, (D) Blank NP and (E) ISCurNP

**
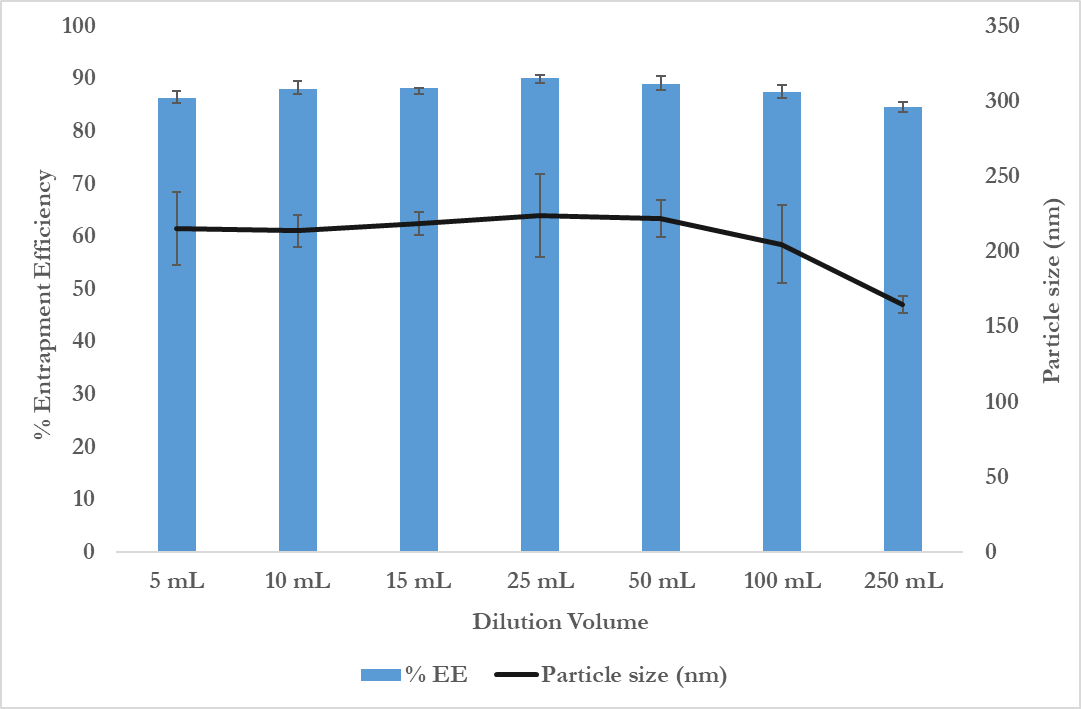
Figure S5:** Effect of dilution volume


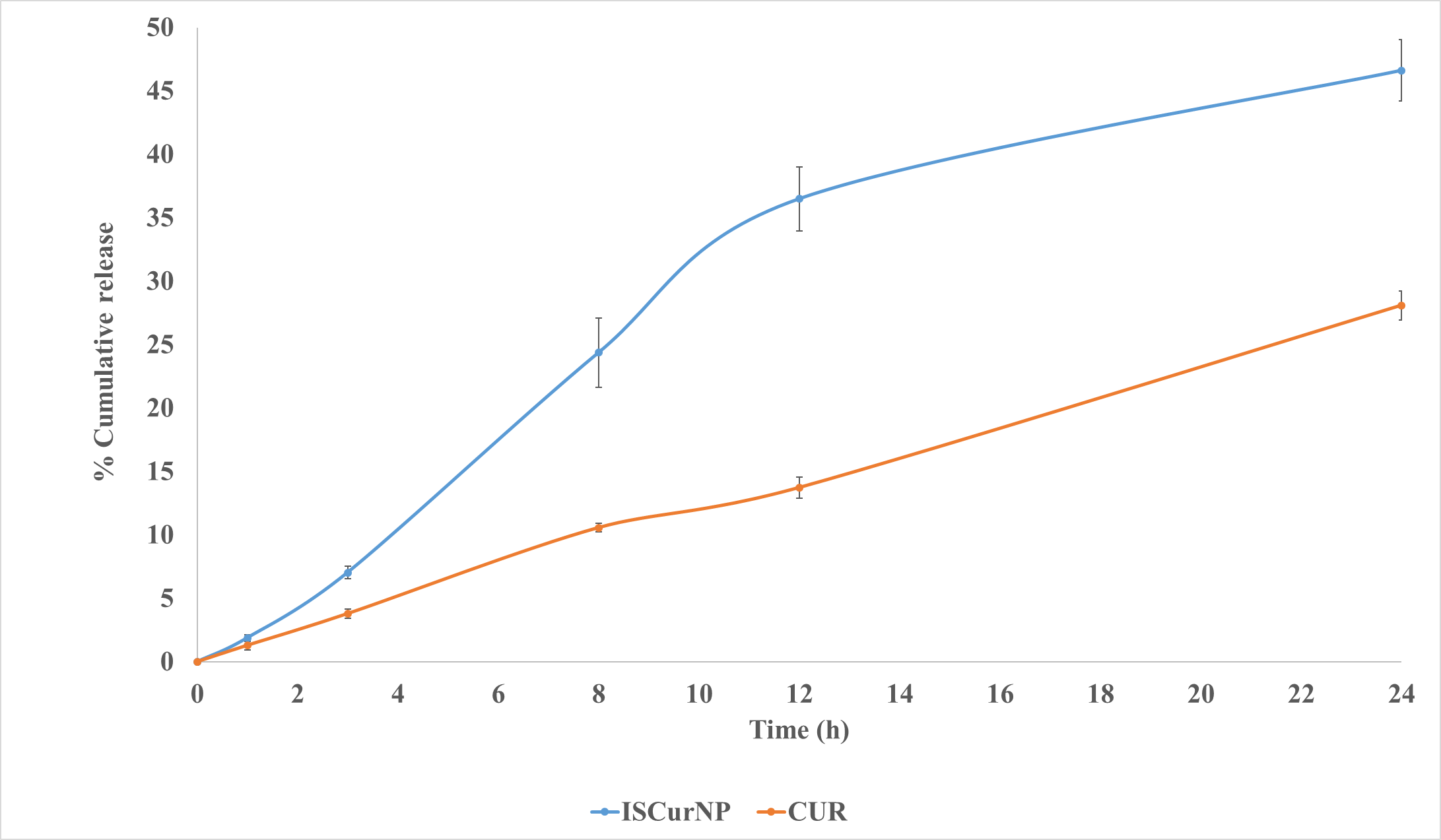


**Figure S6:** Release at physiological buffer pH 6.8

(No release was observed at pH 1.2)


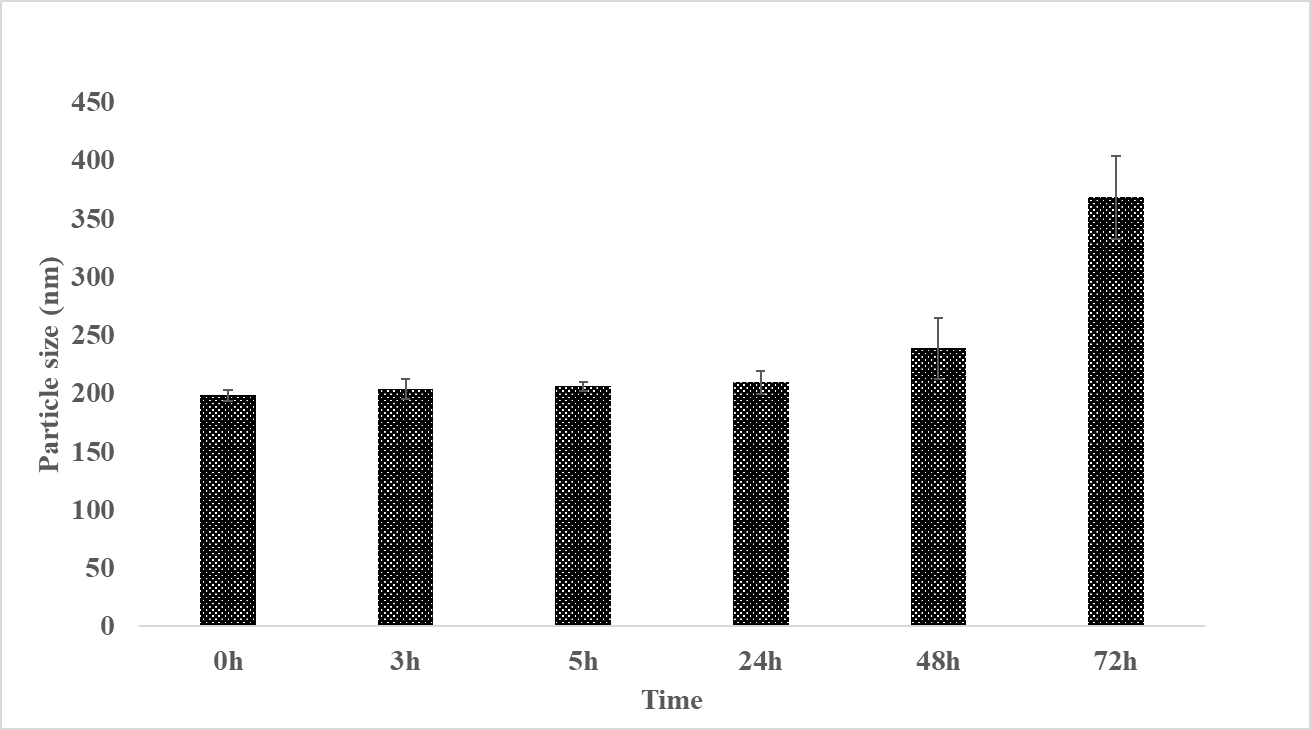
**Figure S7:** Colloidal stability of ISCurNP in DMEM
